# Leu^8^ and Pro^8^ oxytocin agonism differs across human, macaque, and marmoset vasopressin 1a receptors

**DOI:** 10.1038/s41598-019-52024-9

**Published:** 2019-10-29

**Authors:** Aaryn Mustoe, Nancy A. Schulte, Jack H. Taylor, Jeffrey A. French, Myron L. Toews

**Affiliations:** 1 Department of Psychology, Callitrichid Research Center, University of Nebraska at Omaha, Omaha, NE USA; 2 Department of Pharmacology and Experimental Neuroscience, University of Nebraska Medical Center, Omaha, NE USA

**Keywords:** Calcium signalling, Neuroscience

## Abstract

Oxytocin (OXT) is an important neuromodulator of social behaviors via activation of both oxytocin receptors (OXTR) and vasopressin (AVP) 1a receptors (AVPR1a). Marmosets are neotropical primates with a modified OXT ligand (Pro^8^-OXT), and this ligand shows significant coevolution with traits including social monogamy and litter size. Pro^8^-OXT produces more potent and efficacious responses at primate OXTR and stronger behavioral effects than the consensus mammalian OXT ligand (Leu^8^-OXT). Here, we tested whether OXT/AVP ligands show differential levels of crosstalk at primate AVPR1a. We measured binding affinities and Ca^2+^ signaling responses of AVP, Pro^8^-OXT and Leu^8^-OXT at human, macaque, and marmoset AVPR1a. We found that AVP binds with higher affinity than OXT across AVPR1a, and marmoset AVPR1a show a 10-fold lower OXT binding affinity compared to human and macaque AVPR1a. Both Leu^8^-OXT and Pro^8^-OXT produce a less efficacious response than AVP at human AVPR1a and higher efficacious response than AVP at marmoset AVPR1a. These data suggest that OXT might partially antagonize endogenous human AVPR1a signaling and enhance marmoset AVPR1a signaling. These findings aid in further understanding inconsistencies observed following systemic intranasal administration of OXT and provide important insights into taxon-specific differences in nonapeptide ligand/receptor coevolution and behavior.

## Introduction

Oxytocin (OXT) is a nonapeptide neurohormone involved in regulating many critical reproductive and social functions. OXT stimulation leads to contractions of uterine smooth muscle tissue that controls the onset and speed of labor, and OXT facilitates the milk-letdown release in mammary tissue for lactation^1^. OXT signaling in the brain is also necessary for regulating motivation for parental, social, and sexual behavior^2–4^. OXT is produced by neurons in the hypothalamic paraventricular and supraoptic nuclei, and OXT neurons are found throughout regions of the ‘social brain’^5,6^. Differences in the expression of OXT receptors (OXTR) are thought to be important for species- and individual-level variability in social functioning in primates and rodents^7^, and regulation of OXT signaling is known to have therapeutic benefits in a variety of psychiatric disorders^8^.

The nonapeptide hormone family is present in nearly all animal lineages^9,10^, and OXT-like nonapeptides vary in structure across phyla at the second, third, fourth, or eighth amino acid (AA) position^11^. Specifically in mammals OXT plays a central role in reproduction and parental care, and the prevailing belief was that the OXT molecule was strictly conserved across mammals^12^. However recent discoveries revealed that primates, particularly the primate parvorder Platyrrhini (“New World Monkeys”), constitute an unusual ‘hot-spot’ in OXT structural variability within mammals^13^. Genetic analyses across a broader sample of primate taxa confirmed that the OXT ligand has undergone multiple non-synonymous mutations in the coding region of the OXT gene leading to seven confirmed OXT ligand variants across Platyrrhini primates^14–16^. Furthermore, the genetic variability in OXT and its corresponding G-protein coupled receptor, OXTR, are significantly associated with positive selection for traits including social monogamy and litter size^15,16^. Interestingly, social monogamy and biparental/cooperative infant care are two OXT-dependent behaviors that are disproportionately overrepresented in Platyrrhini primates relative to other primate and mammalian clades^11,17^.

OXTR also show high structural variability across primates, particularly in the N-terminus of the receptor^15,16^, which is the receptor region necessary for binding to the tail of the OXT ligand^18,19^. There is strong evidence for coevolution between OXT and its OXTR^10,15,16^, suggesting that these changes in the OXT ligand are likely consequential for receptor functioning. One tantalizing explanation for this coevolution is that a change in the OXT molecule from the ancestral mammalian Leu^8^-OXT to Pro^8^-OXT (Pro^8^ being the most prevalent AA substitution from the ancestral OXT) results in a significant structural alteration in the 8th AA position. This change causes a ‘bent tail’ in the ligand that leads it to be more sterically constrained, and this tail portion of the OXT molecule is critical for OXT receptor binding and activation^18,20–22^.

Whether these evolutionary changes in the OXT ligand lead to important functional differences is an important question. Overall, it appears that Pro^8^-OXT usually produces stronger behavioral and pharmacological outcomes than Leu^8^-OXT. When given exogenously in controlled behavioral tests, both Pro^8^-OXT and Leu^8^-OXT ligands can modulate social behavior, but Pro^8^-OXT often produces stronger effects^11,14^. Specifically, in marmosets (*Callthrix* spp.), a mostly socially monogamous and biparental primate that endogenously express Pro^8^-OXT, Pro^8^-OXT is more effective at modulating mate and stranger-directed behaviors than the ancestral Leu^8^-OXT ligand. Interestingly, even in rats, a species where males are not parental and parental behavior is OXT-dependent, Pro^8^-OXT was more effective at inducing parental effort from males than Leu^8^-OXT^23^. Pharmacological evidence also supports the notion that Pro^8^-OXT is more efficacious and potent than Leu^8^-OXT at primate OXTR. Pro^8^-OXT binds with higher affinity at primate OXTR (in both Pro^8^-OXT and Leu^8^-OXT expressing primates) and produces greater calcium signaling responses compared with Leu^8^-OXT^24,25^, but others have reported minimal signaling differences^23^. These data strongly suggest these evolutionary changes in the OXT ligand lead to important functional consequences presumably through OXT activation of OXTR.

Although OXT signaling primarily occurs through the activation of OXTR, particularly via G_q_-mediated Ca^2+^ mobilization and downstream signaling^23,26^, OXT also alters physiological and behavioral function by activating receptors of the closely related ‘sister’ nonapeptide arginine vasopressin (AVP)^23,27,28^. OXT activation of vasopressin 1a receptors (AVPR1a) can produce full or partial G_q_-mediated Ca^2+^ signaling responses and can alter receptor desensitization and internalization^23^. OXT activation of AVPR1a leads to diverse behavioral outcomes^29^, and the AVP system has been strongly implicated as an important neural regulator in neurodevelopmental disorders including Autism Spectrum Disorders (ASD)^30,31^. Given the broad biological functions of OXT and AVP receptor signaling, and the fact that these OXT and AVP nonapeptides only differ at AA positions 3 and 8, AA substitutions in OXT-like ligands may differentially impact the degree of OXT ligand agonism across primate AVPR1a as they do at OXTR.

Because there is considerable crosstalk between OXT and AVP and their canonical receptors, we examined whether binding affinities, signaling potencies, and signaling efficacies of OXT isoforms varied across primate AVPR1a, with each OXT isoform showing differential levels of agonism at species-specific AVPR1a compared to ‘maximal’ AVP-induced responses. If these evolutionary changes in the OXT ligand structure correspond to functional alterations in the pharmacological properties of both OXTR and AVPR1a signaling across primates, these findings, in turn, may provide important insights into the taxon-specific and receptor-specific patterns underlying the OXT- and/or AVP-dependent modulation of social behavior in primates.

## Results

### ^125^I-OVTA competition binding with OXT and AVP at primate AVPR1a

Saturation binding assays were performed to explore species-level differences in binding affinities for the antagonist radioligand ^125^I-OVTA to AVPR1a (SI Fig. 1). B_max_ values (i.e., maximal binding) for human, macaque, and marmoset ranged from ~6.21–12.71 fmol/well with marmosets AVPR1a CHO clone showing the highest B_max_ values (Table 1). Overall, human, marmoset, and macaque AVPR1a had relatively similar *K*_*d*_ values, ranging from 331–1165 pM (Table 1). The affinities for AVPR1a were also similar to those for ^125^I-OVTA at human, macaque, and marmoset OXTR (161–481 pM) published previously (Taylor *et al*., 2018). These findings confirm that there are only relatively small differences in binding affinities for ^125^I-OVTA among AVPR1a from these three primate species.

**Table 1:** Binding affinities for ligands at primate AVPR1a.

AVPR1a	*K*_*i*_			^125^I-OVTA		Rank Order Potency
	AVP	Leu^8^-OXT	Pro^8^-OXT	*B*_*max*_ (fmol/well)	*K*_*d*_ (pM)	
Human	0.63 ± 0.24	15.78 ± 0.33	8.71 ± 0.37	6.21	1165	AVP > Leu^8^ = Pro^8^
Macaque	1.21 ± 0.10	30.02 ± 0.16	23.77 ± 0.17	8.50	331	AVP > Leu^8^ = Pro^8^
Marmoset	0.86 ± 0.02	247 ± 0.06	175.9 ± 0.05	12.71	944	AVP > Leu^8^ = Pro^8^

IC50 presented as nM ± Std. Error. Efficacy is presented as % maximum AVP response ± Std. Error. Human (n = 3); Macaque (n = 3); Marmoset (n = 5). Leu^8^ = Leu^8^-OXT; Pro^8^ = Pro^8^-OXT.

**Figure 1:**
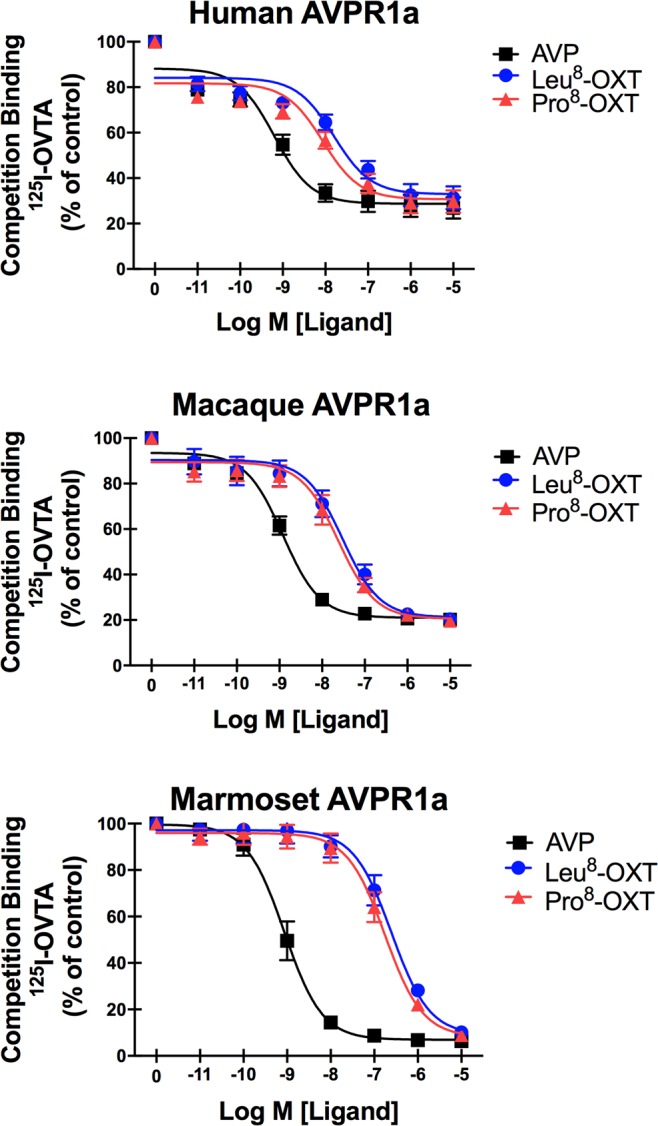
Competition Binding of ^125^I-OVTA with OXT and AVP at Primate AVPR1a. Binding competition curves for AVP, Pro^8^-OXT, and Leu^8^-OXT for each of the primate AVPR1a. Increasing concentrations of competitor ligand (OXT, AVP) were added to fixed concentration of ^125^I-OVTA in intact CHO cells expressing one of the primate AVPR1a. All values are expressed as percentage of maximal (control) binding in the absence of OXT or AVP.

In competition binding assays with ^125^I-OVTA, AVP exhibited higher binding affinity to all primate AVPR1a than did either the Leu^8^-OXT or Pro^8^-OXT ligands, and Leu^8^-OXT and Pro^8^-OXT did not significantly differ from each other in affinity (K_i_ value) at any of the primate AVPR1a (Fig. 1). At human AVPR1a there was a significant difference in competitive binding affinity among ligands [*F*(2,9) = 45.17, *p* < 0.001]. AVP displayed a significantly higher affinity (0.6 nM) than both Leu^8^-OXT (15.8 nM, *p* < 0.001) and Pro^8^-OXT (8.7 nM, *p* < 0.001), but Leu^8^-OXT and Pro^8^-OXT did not differ from each other (*p* = 0.26). At macaque AVPR1a there was a significant difference in binding affinity among ligands [*F*(2,6) = 52.99, *p* < 0.001]. AVP displayed significantly higher affinity (1.2 nM) than both Leu^8^-OXT (30.0 nM, *p* < 0.001) and Pro^8^-OXT (23.8 nM, *p* < 0.001), but Leu^8^-OXT and Pro^8^-OXT did not differ from each other (*p* = 0.77). Finally, at marmoset AVPR1a there was also a significant difference in binding affinity among ligands [*F*(2,6) = 119.1, *p* < 0.001]. AVP displayed a significantly higher affinity (0.9 nM) than both Leu^8^-OXT (247 nM, *p* < 0.001) and Pro^8^-OXT (176 nM, *p* < 0.001), but Leu^8^-OXT and Pro^8^-OXT did not differ from each other (*p* = 0.69). Interestingly, though AVP affinities were similar for all primate AVPR1a, both Leu^8^-OXT and Pro^8^-OXT had affinities ~10 fold lower at marmoset AVPR1a compared to macaque and human AVPR1a (Table 1).

### OXT and AVP stimulation of Ca^2+^ signaling at primate AVPR1a

**Figure 2:**
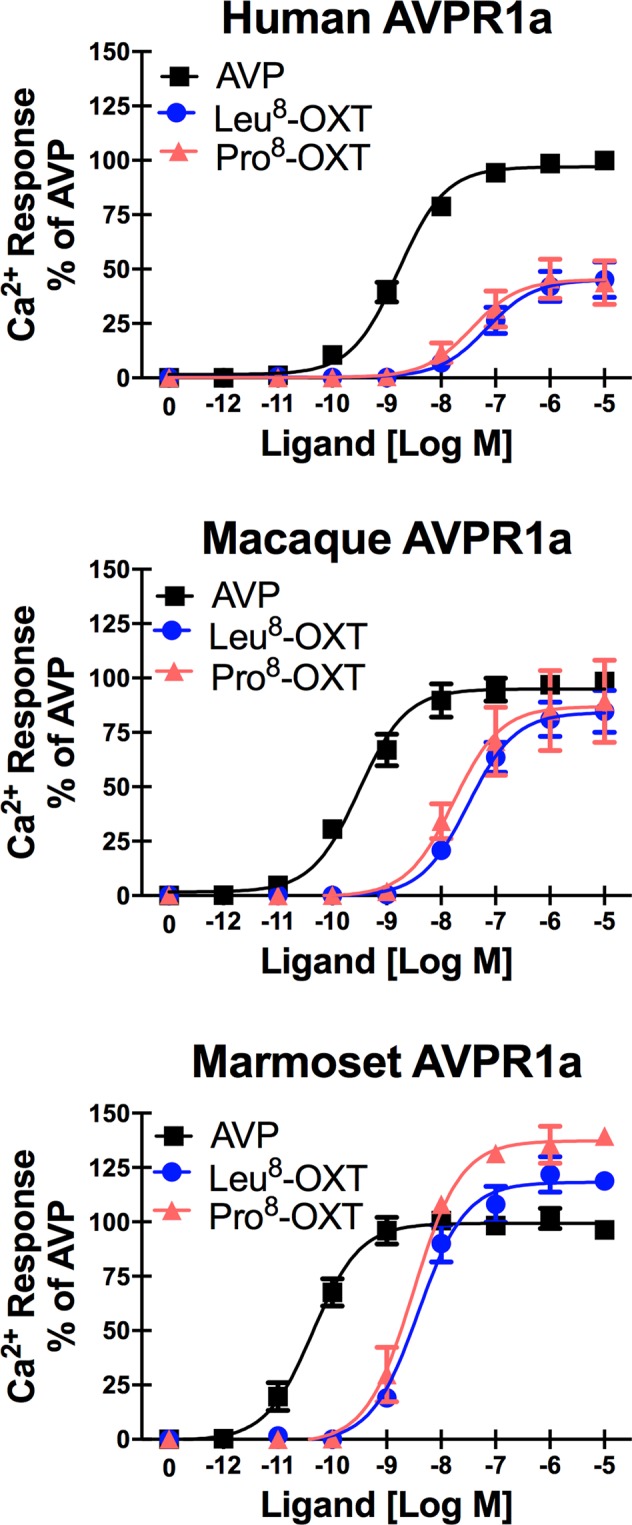
OXT and AVP Stimulation of Ca^2+^ Signaling at Primate AVPR1a. Intracellular Ca^2+^ responses in CHO cells expressing each of the primate AVPR1a in response to stimulation with varying concentrations of AVP, Pro^8^-OXT, or Leu^8^-OXT. All values are expressed as the percentage of the maximal AVP (10^−5^ M) Ca^2+^ response for each primate species AVPR1a.

Both Leu^8^-OXT and Pro^8^-OXT are able to activate Ca^2+^ mobilization in AVPR1a, though in all species the rank order of potencies (Ca^2+^ EC_50_ values) for OT were significantly lower than for AVP at AVPR1a, and Leu^8^-OXT and Pro^8^-OXT were equipotent at all three primate AVPR1a (Fig. 2 and Table 2). At human AVPR1a there was a significant difference in Ca^2+^ response potency among ligands [*F*(2,15) = 49.99, *p* < 0.001]. AVP was significantly more potent (1.6 nM) at producing a Ca^2+^ response than both Leu^8^-OXT (69.3 nM, *p* < 0.001) and Pro^8^-OXT (36.2 nM, *p* < 0.001), but Leu^8^-OXT and Pro^8^-OXT did not differ from each other (*p* = 0.72). At macaque AVPR1a, there was a significant difference in Ca^2+^ response potency among ligands [*F*(2,6) = 140.0, *p* < 0.001]. AVP was significantly more potent (0.3 nM) at producing a Ca^2+^ response than both Leu^8^ -OXT (31.4 nM, *p* < 0.001) and Pro^8^-OXT (17.0 nM, *p* < 0.001), with no difference in potency between Leu^8^-OXT and Pro^8^-OXT (*p* = 0.20). The same pattern was also observed at marmoset AVPR1a (AVP = 0.1 nM, Leu^8^-OXT = 3.8 nM, Pro^8^-OXT = 3.14 nM) [*F*(2,6) = 61.91, *p* < 0.001].

**Table 2:** Ca^2+^ mobilization potencies and efficacies for OXT/AVP at primate AVPR1a.

AVPR1a	Ca^2+^ EC50			Ca^2+^ Response Efficacy (% Max AVP)		Rank Order Potency	Rank Order Efficacy
	AVP	Leu^8^-OXT	Pro^8^-OXT	Leu^8^-OXT	Pro^8^-OXT		
Human	1.64 ± 0.05	69.34 ± 0.18	36.20 ± 0.25	45.14 ± 3.32	45.21 ± 4.06	AVP > Leu^8^ = Pro^8^	AVP > Leu^8^ = Pro^8^
Macaque	0.31 ± 0.09)	31.40 ± 0.11	17.02 ± 0.22	84.21 ± 3.25	86.93 ± 6.65	AVP > Leu^8^ = Pro^8^	AVP = Leu^8^ = Pro^8^
Marmoset	0.05 ± 0.05	3.82 ± 0.05	3.14 ± 0.05	118.20 ± 3.41	137.40 ± 2.75	AVP > Leu^8^ = Pro^8^	AVP < Leu^8^ < Pro^8^

EC50 presented as nM ± Std. Error. Efficacy is presented as % maximum AVP response ± Std. Error. Human (n = 6); Macaque (n = 3); Marmoset (n = 3).

Though there were no differences in OXT potency (dose needed to produce 50% maximal response, EC50) at AVPR1a, there were significant species and ligand differences in the efficacy (maximal magnitude response) of OXT agonism at human and marmoset AVPR1a. At human AVPR1a, OXT functioned as a partial agonist compared to the canonical ligand AVP [*F*(2,15) = 18.99, *p* < 0.001]. Both Leu^8^-OXT and Pro^8^-OXT produced an approximately 45% maximal Ca^2+^ response compared to AVP (*p* < 0.001). Conversely, at marmoset AVPR1a, OXT functioned as a superagonist, generating a larger maximal response compared to the canonical ligand AVP [*F*(2,6) = 6.03, *p* < 0.05]. Specifically, the Leu^8^-OXT maximal response was 118% of that for AVP (*p* < 0.001), and the Pro^8^-OXT maximal response was 137% of that for AVP (*p* < 0.001). Compared to Leu^8^-OXT, Pro^8^-OXT produced a greater maximal Ca^2+^ response at AVPR1a than Leu^8^-OXT (*p* < 0.05). For macaque AVPR1a, AVP, Leu^8^-OXT, and Pro^8^-OXT all produced equal maximal Ca^2+^ responses [*F*(2,6) = 0.23, *p* = 0.80].

### OXT partial antagonism of AVP Ca^2+^ mobilization at human and marmoset AVPR1a

**Figure 3:**
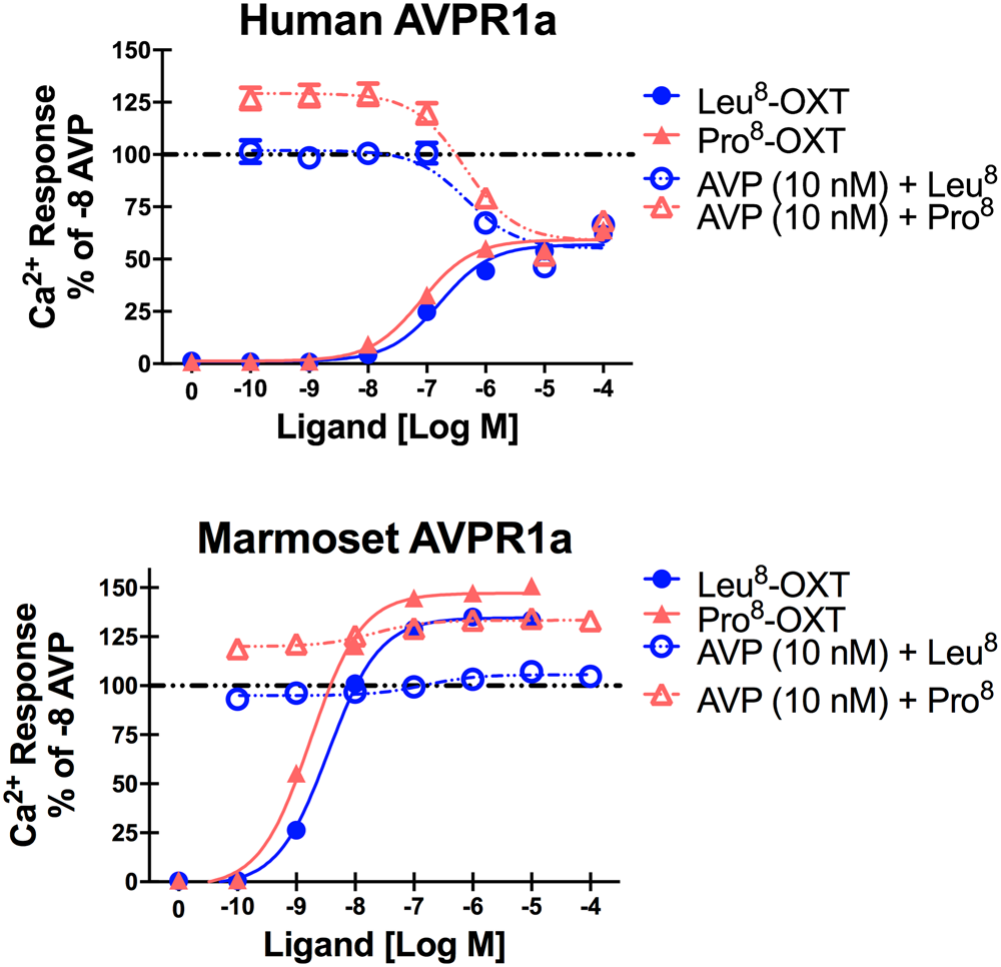
OXT Partial Antagonism of AVP Ca^2+^ Mobilization at Human and Marmoset AVPR1a. Intracellular Ca^2+^ responses in CHO cells expressing each of the primate AVPR1a in response to stimulation with varying concentrations of Pro^8^-OXT or Leu^8^-OXT in the presence or absence of 10^−8^ M AVP (10 nM). All values are expressed as the relative percentage of the AVP (10^−8^ M) Ca^2+^ response for each primate species AVPR1a.

Partial agonists should also exhibit partial antagonism. To confirm this, Leu^8^-OXT and Pro^8^-OXT were coadministered with 10 nM AVP (a concentration of AVP that alone produces approximately 80% of the maximal Ca^2+^ response) for both human and marmoset AVPR1a. For human AVPR1a, both Leu^8^-OXT and Pro^8^-OXT caused a concentration-dependent decrease in AVP-stimulated Ca^2+^ mobilization. Both Leu^8^-OXT and Pro^8^-OXT at concentrations < 1 μM with and without coadministration of 10 nM AVP produced only 50% of the maximal Ca^2+^ mobilization response to AVP, confirming that both OXT ligands act as both partial agonists and partial antagonists at human AVPR1a (Fig. 3). For marmoset AVPR1a, OXT did not function as a partial antagonist of AVP, (i.e., OXT did not induce inhibition of the Ca^2+^ signaling response when co-administered with AVP) (Fig. 3). Interestingly, in both human and marmoset AVPR1a, Pro^8^-OXT coadministered with AVP always produced a greater maximal response than Leu^8^-OXT when coadministered with AVP. This occurred even at concentrations of OXT that did not produce Ca^2+^ responses on their own. OXT partial antagonism experiments were not performed in macaque AVPR1a because OXT did not partially agonize or superagonize Ca^2+^ responses at macaque AVPR1a.

### Coupling efficiency at primate AVPR1a

We measured a simplified form of coupling efficiency as a ratio of the concentration of ligand needed to mobilize Ca^2+^ responses in primate AVPR1a (potency/EC50) relative to the ligand binding affinity (K_i_) at AVPR1a (Table 3). This metric provides insight into whether OXT/AVP ligands produce equal signaling responses across different receptors in the presence or absence of unbound/spare receptors, i.e., a extra receptors than what is required to produce a maximal response^32^. Only the marmoset AVPR1a showed high coupling efficiency, with EC_50_ values that were about 1.8 log units higher than their K_D_ values for both Leu^8^-OXT and Pro^8^-OXT, respectively. Conversely, human AVPR1a showed negative coupling efficiency values, suggesting that human AVPR1a require relatively more AVPR1a to produce Ca^2+^ responses from OXT ligands compared to marmoset AVPR1a. Macaque AVPR1a coupling efficiencies for AVP, Leu^8^-OXT, and Pro^8^-OXT showed minimal difference (less than one log unit) compared to those for human and marmoset AVPR1a.

**Table 3:** Coupling efficiencies for ligands at primate AVPR1a.

	AVP	Leu^8^-OXT	Pro^8^-OXT
Human	−0.42	−0.64	−0.62
Macaque	0.59	−0.02	0.15
Marmoset	1.28	1.81	1.75

Calculated as a Potency/Affinity Ratio [−Log(Ca^2+^EC50/*K*_*i*_)].

## Discussion

This study is the first to evaluate potential differences in Leu^8^-OXT and Pro^8^-OXT binding affinity, Ca^2+^ signaling potency and efficacy, OXT partial antagonism, and receptor coupling efficiency across a variety of primate AVPR1a. Previous studies have evaluated whether the documented coevolutionary changes in OXT ligands and OXTR in Platyrrhini primates would produce demonstrable and unique properties for OXT-OXTR signaling in these species^23–25^. These studies found that changes to the OXT molecule, namely the Leu^8^ to Pro^8^ AA substitution in OXT, produced only modest changes in binding and signaling across primate OXTR. OXT exhibits a significant degree of ‘cross-talk’ with AVP receptors (primarily AVPR1a), and OXT and AVP exhibit potential overlap in behavioral outcomes via OXT and AVP signaling in the brain. It is therefore plausible that OXT modifications would lead to a functional selective advantage through differences in OXT interactions with AVPR1a. The data from this study support three key conclusions: (1) AVP binds with significantly higher affinity than OXT at human, marmoset, and macaque AVPR1a, and marmoset receptor AVPR1a show a 10-fold lower OXT binding affinity compared to human and macaque AVPR1a. (2) There are no significant differences in binding affinity or Ca^2+^ signaling potency between Leu^8^-OXT and Pro^8^-OXT at primate AVPR1a. (3) Both OXT isoforms exhibit differential levels of agonism/antagonism across primate AVPR1a, acting as partial agonists and partial antagonists at human AVPR1a and as superagonists at marmoset AVPR1a.

The idea that differences in the OXT ligand structure would result in functional differences in primate AVPR1a binding and/or signaling properties was only partially supported. While Leu^8^-OXT and Pro^8^-OXT showed no differences in binding affinity or Ca^2+^ mobilization potencies at any of the primate AVPR1a, there was a significant difference in levels of OXT agonism both across primate AVPR1a and between OXT variants. Pro^8^-OXT produced a significantly higher maximal response compared to Leu^8^-OXT at marmoset AVPR1a. These pharmacological findings also partially align with previous work examining effects of Leu^8^-OXT and Pro^8^-OXT signaling at OXTR. Pro^8^-OXT exhibited modestly higher potencies than Leu^8^-OXT at primate OTRs^25^; Pro^8^-OXT produced more efficacious Ca^2+^ responses at marmoset OXTR but not at human OXTR^24^; and Pro^8^-OXT produced lower recruitment of β-arrestin and less receptor desensitization and internalization at both human OXTR and AVPR1a, where only human receptors were tested^23^. Perhaps the most compelling finding from this study was that OXT exhibits differential agonism at human and marmoset AVPR1a. Similar to OXT signaling at OXTR, Pro^8^-OXT was more efficacious than Leu^8^-OXT at marmoset but not human AVPR1a. Previous pharmacological studies of marmoset OXTR did not explicitly test if different OXT ligands were partial agonists at marmoset and human OXTR^24^, and that study did not make direct comparisons of OXT agonism to AVP agonism at OXTR. However, AVP appears to be a full agonist relative to both Leu^8^-OXT and Pro^8^-OXT at primate OXTR based on data reported across human, marmoset, macaque, and titi monkey OXTR^25^.

The observation that Leu^8^-OXT and Pro^8^-OXT act as a partial agonists/antagonists at human AVPR1a is a novel finding. The first reported study evaluating the pharmacological profile of Pro^8^-OXT at human AVPR1a showed that Pro^8^-OXT is a full agonist at producing Ca^2+^ responses compared to AVP, while Pro^8^-OXT was only a partial agonist for β-arrestin recruitment at both human AVPR1a or OXTR^23^. It is unclear what underlies the difference in Ca^2+^ responses from the human AVPR1a tested in this study and the human AVPR1a tested previously^23^. Based on the clear partial agonism at human AVPR1a, we tested whether, as expected, OXT also functioned as a partial antagonist of the AVP Ca^2+^ response at both human and marmoset AVPR1a. Adding either OXT isoform along with AVP reduced the AVP Ca^2+^ response, but only for human AVPR1a. This confirms that OXT is a partial antagonist at human AVPR1a but not at marmoset AVPR1a. We further corroborated this finding by testing a marmoset AVPR1a clone with lower receptor expression (as indicated by saturation binding with ^125^I-OVTA), and again both OXT ligands functioned as full agonists with slightly lower potency, with the Pro^8^-OXT response greater than for Leu^8^-OXT, eliminating concerns that species-differences in OXT agonism at AVPR1a were due to different expression levels of AVPR1a across species (SI Fig. 2) and/or differences in AVP signaling efficacy at 10 nM doses across primate AVPR1a. We also observed that all non-maximal OXT doses (<100 nM) of Pro^8^-OXT coadministered with 10 nM AVP at each primate AVPR1a produced a more efficacious agonism than comparable Leu^8^-OXT doses coadministered with 10 nM AVP, even in the presence of OXT doses (<1 nM) that would produce no measurable Ca^2+^ signaling response on their own. The mechanism underlying this finding is currently unclear.

The conclusion that OXT functions as a partial agonist and a partial antagonist for AVP activation of Ca^2+^ signaling responses in human AVPR1a has important implications. Though evidence for endogenously released OXT producing functionally important responses at AVP receptors is limited, some studies have shown that stimulating endogenous OXT release can induce social behavioral responses in rodents via AVPR1a^33,34^. However, a majority of studies that examine the effects of OXT on behavior use exogenous intranasal OXT administration, causing systemic distribution and leading to supraphysiological increases in circulating OXT throughout the periphery. OXT is known to exert dose-dependent behavioral effects^35,36^; thus further studies are warranted to evaluate whether high doses of OXT, in addition to activating OXTR, might also partially antagonize endogenous human AVPR1a signaling, which could aid in further understanding of the inconsistencies observed in behavioral responses following systemic administration of OXT^37^ and the reported “inverted -U-shaped” relationship between OXT dose and behavior^38,39^.

Moreover, differential OXT agonism at AVPR1a could have important implications for understanding the therapeutic potential of nonapeptide treatments in alleviating symptoms associated with neurodevelopmental disorders such as autism spectrum disorders (ASD). For instance, animal models of nonapeptide signaling may not generalize in a simple way to human clinical trials. The impact of intranasal OXT on behaviorally relevant clinical outcomes has shown mixed support in the literature^40,41^, but recent evidence has shown that peripheral use of both a highly selective AVPR1a antagonist and intranasal AVP administration has markedly improved behavioral outcomes for individuals with ASD^30,31^. These findings are important given that similar OXT treatment strategies for ASD have shown mixed efficacy^42,43^, and high doses of OXT could even mitigate potential therapeutic benefits of AVPR1a activation in ASD and surely other behavioral contexts as well. Whether the partial agonism/antagonism at human AVPR1a explains these anomalies merits further study.

It is also noteworthy that differences in OXTR and AVPR1a functioning are important for our broader understanding of the coevolution of nonapeptide signaling system in Platyrrhini primates. While OT acted as a partial agonist at human AVPR1a, OXT (both Leu^8^-OXT and Pro^8^-OXT) instead acted as a superagonist at marmoset AVPR1a. This is especially important from an evolutionary context because the Callitrichid clade has evolved widespread Pro^8^-OXT expression of the OXT ligand, and the Pro^8^ ligand produces stronger behavioral effects^44,45^, potency and efficacy effects at marmoset OXTR^24,25^, and efficacy effects at marmoset AVPR1a (this study). It is unclear whether the higher agonism and coupling efficiency of OXT at marmoset AVPR1a is an important or conserved mechanism underlying the potential coevolution between OXTR and AVPR1a variability with socially monogamous phenotypes in primates^15,46^. More pharmacological and behavioral work utilizing Pro^8^-OXT and other OXT ligands is needed across a broader sampling of primates. Such examples include Leu^8^-OXT expressing titi monkeys that are viewed as socially monogamous and biparental and Pro^8^-OXT expressing primates such as capuchins or squirrel monkeys that are highly social but non-monogamous/biparental. These data combined with the important data published on OXTR and AVPR1a central expression profiles in marmoset, titi, macaque, and humans^7^ would serve as a powerful tool to begin utilizing and targeting diverse non-human primate models of nonapeptide regulation of social behavioral phenotypes.

Clearly, there are many contributing factors to the ways in which OXT and AVP regulate physiological and behavioral outcomes across primates, the relative roles of OXTR and AVPR1a activation, and how well these and other *in vitro* findings translate directly to neural transmission and ultimately behavioral modulation. These relationships are difficult to ascertain, especially in light of currently limited access to primate neural tissue and primate gene-editing techniques. An important first step is to evaluate whether the pharmacological and physiological findings and principles already established for nonapeptide biology in rodents are divergent or conserved across diverse nonhuman primate species. Our findings will serve as a roadmap to target specific pharmacological and physiological properties that may underlie species- or individual-level differences in behavioral and social phenotypes. Behavioral studies have been at the forefront of this effort and have elucidated many key findings about how OXT regulates social behavior in nonhuman primates^14,47–49^, but many of these studies have yet to identify specific neural mechanisms underlying these behavioral effects. Overall, the findings from this study provide important molecular insights into species-level differences in nonapeptide ligand/receptor coevolution and ‘cross-talk’ between OXT and AVP.

## Methods

### Primate AVPR1a transfection and cell culture

Chinese hamster ovary (CHO; Female origin) cells were purchased from American Type Culture Collecton (Manassas, VA) and cultured at 37 ^◦^C with 5% CO_2_ using Ham’s F12 medium supplemented with 10% fetal bovine serum and 100 units/mL penicillin and 100 µg/ml streptomycin. Human, marmoset, and macaque AVPR1a plasmids were purchased from Genscript (Piscataway, NJ) in a pcDNA3.1+ vector based on confirmed genetic sequences. CHO cells were transfected using Turbofect according to the manufacturer’s instructions and were kept under selective pressure using 400 µg/mL G418 antibiotic. Individual clonal lines were generated from batch-transfected cells by plating approximately 10 cells/mL (1 cell/100 µL) into 96-well plates. Clonal lines that originated from a single colony were screened using an intact cell ^125^I-ornithine vasotocin analog (^125^I-OVTA) binding assay and selected for similar receptor expression across species, defined as specific radioligand binding. CHO cells showed no endogenous OXTR and AVPR1a binding or signaling activity in response to OXT and/or AVP ligands (SI Fig. 3).

### Intact cell saturation binding assays

CHO cells expressing primate AVPR1a were plated at 150,000 cells/mL (15,000 cells per well/100 µL) into 96-well plates and incubated at 37 °C for 48 hours to achieve 80–90% confluence. On the day of assay, growth medium was aspirated and cells were quickly washed once with 100 µL ice-cold high glucose HEPES-buffered Dulbecco’s Modified Eagle’s Medium containing 0.1% bovine serum albumin (HGH-BSA) and then placed on ice. 50 µL of ice-cold HGH-BSA containing ^125^I-OVTA (PerkinElmer) in doubling concentrations (~15 to 2000 pM) was added in triplicate (technical replicates) and incubated for 3 hours on ice. 3 hours is the minimum incubation time on ice for ^125^I-OVTA and ^125^I-OVTA + AVP/OXT to reach equilibrium (SI Fig. 4) in CHO cells transfected with human and marmoset AVPR1a. Cells were washed four times with 100 µL ice-cold HGH-BSA, solubilized with 100 µL 0.2 N NaOH, and radioactivity quantified with a gamma counter. We also counted aliquots of the used binding medium (i.e., free ^125^I-OVTA) to quantify free radioligand CPM directly, eliminating any concerns about differential depletion of ligand due to differential receptor expression levels across species or time. Non-specific binding was defined as ^125^I-OVTA binding occurring in the presence of excess competitor (10^−4^ M AVP). Binding affinity for ^125^I-OVTA was determined after correcting for non-specific binding by plotting specific bound/free vs. bound using a single-site binding equation (Graphpad Software Inc., La Jolla, CA). Assays were done at least three times on three different days using fresh aliquots of ^125^I-OVTA and competitor, and *K*_*d*_ values were averaged across at least three biological replicates.

### Intact cell competition binding assays

CHO cells expressing primate AVPR1a were plated at 150,000 cells/mL (15,000 cells per well/100 ul) into 96-well plates and incubated at 37 °C for 48 hours/grown to 80–90% confluence. On the day of assay, growth medium was aspirated and cells were quickly washed once with 100 µL ice-cold HGH-BSA and then placed on ice. Then 50 µL of ice-cold HGH-BSA containing ~50,000 CPM ^125^I-OVTA were added in triplicate (technical replicates) to all wells in the presence or absence of 10^−11^ to 10^−5^ M Pro^8^-OXT (CYIQNCPPG-NH2; Anaspec), Leu^8^-OXT (CYIQNCPLG-NH2; Anaspec) or AVP (CYFQNCPRG-NH2; Anaspec), and incubated for three hours on ice. Cells were washed four times with 100 µL ice-cold HGH-BSA, solubilized with 100 µL 0.2 N NaOH, and radioactivity was quantified with a gamma counter. Half-maximal inhibitory concentrations (IC_50_) were determined by plotting bound ^125^I-OVTA vs. competitor concentration. IC_50_ values were then corrected using the Cheng-Prusoff equation with each receptor’s K_D_ for ^125^I-OVTA to produce *K*_*i*_ values for the competing ligands. Assays were done at least three times on three different days using fresh aliquots of ^125^I-OVTA and Leu^8^-OXT, Pro^8^-OXT, and AVP with at least three biological replicates per clone.

### Ca^2+^ mobilization assays

CHO cells expressing primate AVPR1a were plated at 150,000 cells/mL (15,000 cells per well/100 µL) into 96-well plates and incubated at 37 °C for 48 hours/grown to 80–90% confluence. On the day of assay, growth medium was aspirated and cells were incubated at 37 °C with 100 µL Fluo-4 Direct dye mixed in Fluo-4 Direct Ca^2+^ Assay Buffer with 5 mM probenecid for ~45 minutes. Using a FlexStation 2 (Molecular Devices), baseline fluorescence was measured at 37 °C followed by stimulated fluorescence in the presence or absence of 10^−12^ to 10^−5^ M Pro^8^-OXT, Leu^8^-OXT, or AVP (3 × technical replicates). Peak fluorescence minus baseline fluorescence was plotted as a function of ligand concentration to determine EC_50_ values. Assays were done at least three times on three different days using fresh aliquots of Leu^8^-OXT, Pro^8^-OXT, and AVP for three biological replicates per clone. We determined the degree of OXT Ca^2+^ agonism/antagonism at AVPR1a by repeating the same procedures for Leu^8^-OXT alone, Pro^8^-OXT alone, and OXT ligands coadministered with 10^−8^ M AVP (10 nM) (3 × technical and biological replicates) with concentrations of OXT from 10^−10^ to 10^−5^ M compared to coadministration of OXT concentrations from 10^−10^ to 10^−4^ M together with 10^−8^ AVP.

### Data analyses

Binding affinities for ^125^I-OVTA at each primate AVPR1a were calculated by subtracting nonspecific binding and then plotting bound ^125^I-OVTA *vs*. free ^125^I-OVTA. Because concentrations of ^125^I-OVTA were not identical from experiment to experiment, technical replicates within each experiment (n = 3) were normalized and then corrected using the Cheng-Prusoff equation. Technical replicates were averaged and used as biological replicates (n = 3 per clone) to determine and compare K_i_ values for each ligand within species. Differences in Ca^2+^ mobilization potency (EC_50_) and maximal response to OXT were determined by normalizing OXT-induced (Log M) Ca^2+^ responses as a percentage of maximal (100%) AVP-induced Ca^2+^ response. We averaged across technical replicates (n = 3) within each biological replicate and then averaged across the biological replicates (n = 3), normalized the data, and tested for significant differences of best-fit LogEC_50_ using one-way ANOVA analyses. Post hoc analyses to assess ligand comparisons (Pro^8^-OXT vs. Leu^8^-OXT, Pro^8^-OXT vs. AVP, Leu^8^-OXT vs. AVP) were performed using Tukey’s posthoc test with a Bonferroni-corrected cutoff to determine statistically significant differences in best-fit LogEC_50_. All best-fit data (*K*_*i*_, EC50, and Ca^2+^ maximal responses) were analyzed using the nonlinear least squares curve-fitting capabilities of GraphPad Prism.

## Supplementary information

Supplemental Figures

## Data Availability

Raw data and clonal cell lines (CHO clonal cell lines expressing either human, macaque, or marmoset AVPR1a) are available upon request.
