# Supplementary material for: Leu8 and Pro8 oxytocin agonism differs across human, macaque, and marmoset vasopressin 1a receptors
Source: Sci Rep. 2019 Oct 29;9:15480. doi: 10.1038/s41598-019-52024-9 (PMC6820730; doi:10.1038/s41598-019-52024-9)
Supplement: Supplementary file 1 — Supplemental Figures [file 41598_2019_52024_MOESM1_ESM.docx]

*SUPPLEMENTARY INFORMATION*

**Leu^8^ and Pro^8^ oxytocin agonism differs across human, macaque, and marmoset vasopressin 1a receptors**

Aaryn Mustoe^1,2,*^, Nancy A. Schulte^2^, Jack H. Taylor^1,2^, Jeffrey A. French^1^, Myron L. Toews^2^

^1^Department of Psychology, Callitrichid Research Center, University of Nebraska at Omaha, Omaha, NE USA

^2^Department of Pharmacology and Experimental Neuroscience, University of Nebraska Medical Center, Omaha, NE USA

**
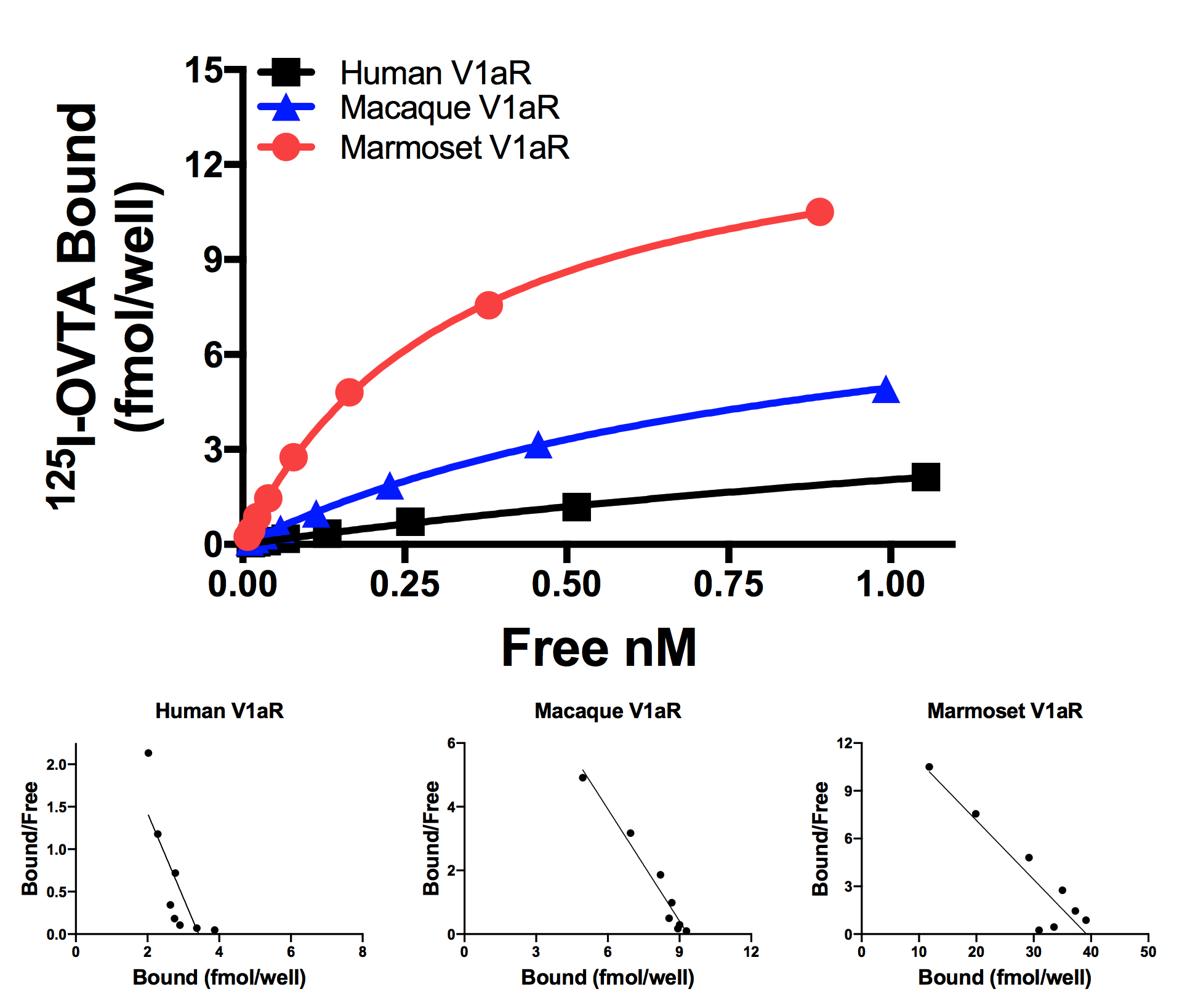
**

**SI Figure 1: Representative saturation assays and scatchard plots for 125I-OVTA binding to primate AVPR1a.** Cells on 96-well plate were incubated on ice in 50 μL of binding medium with the indicated concentrations of 125I-OVTA for 3 hrs. Data are from single experiment with all three receptors tested side by side in triplicate.


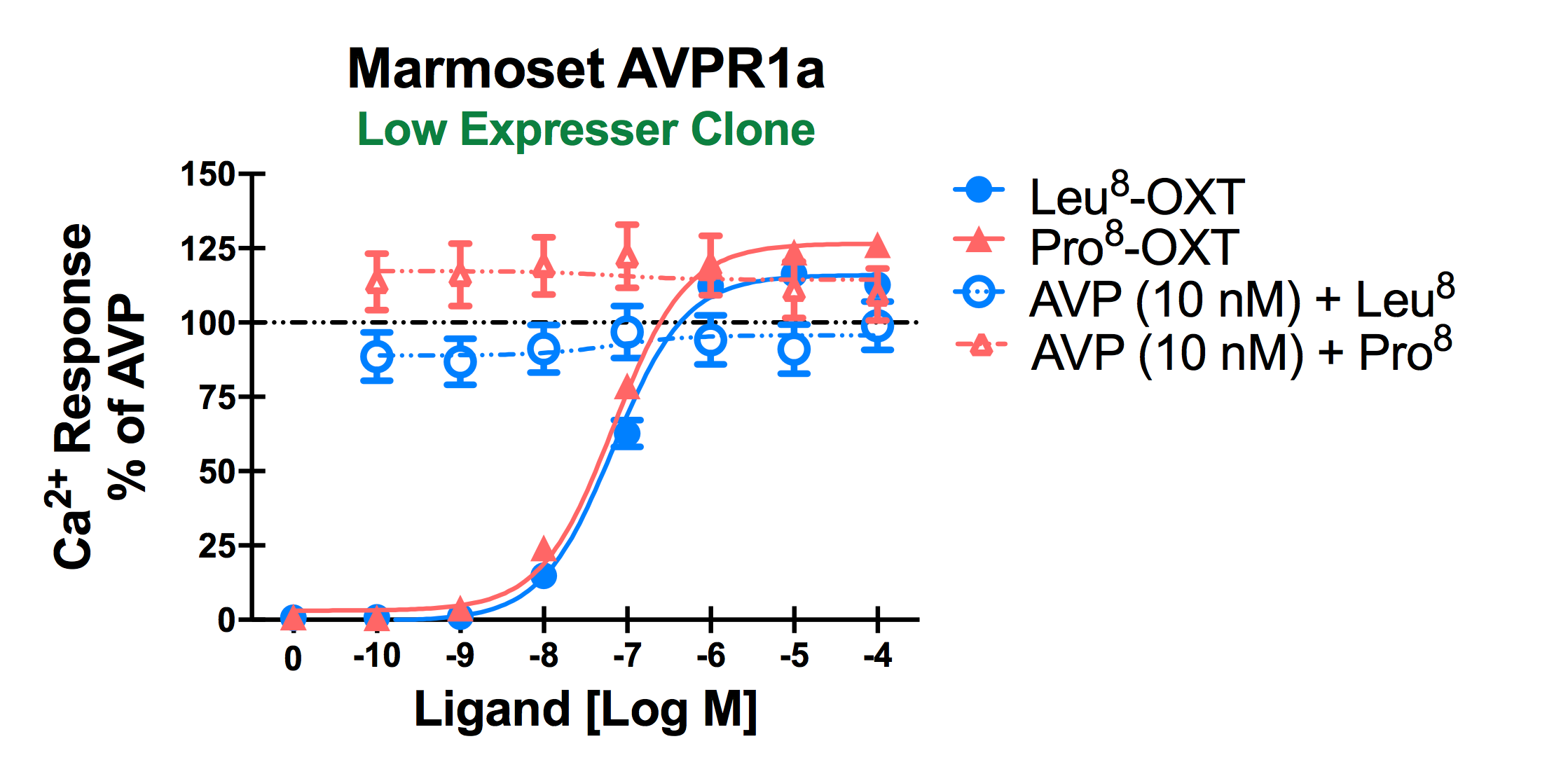


**SI Figure 2**: **Figure 3: OXT partial antagonism of AVP Ca^2+^ mobilization at Low expressing marmoset AVPR1a.** Intracellular Ca^2+^ responses in CHO cells expressing marmoset AVPR1a in response to stimulation with varying concentrations of Pro^8^-OXT or Leu^8^-OXT in the presence or absence of 10^-8^ M AVP (10 nM). All values are expressed as the relative percentage of the AVP (10^-8^ M) Ca^2+^ response for marmoset AVPR1a. These findings demonstrate that OXT superagonism in marmoset AVPR1a cells does not change in conditions of low AVPR1a receptor expression.

**
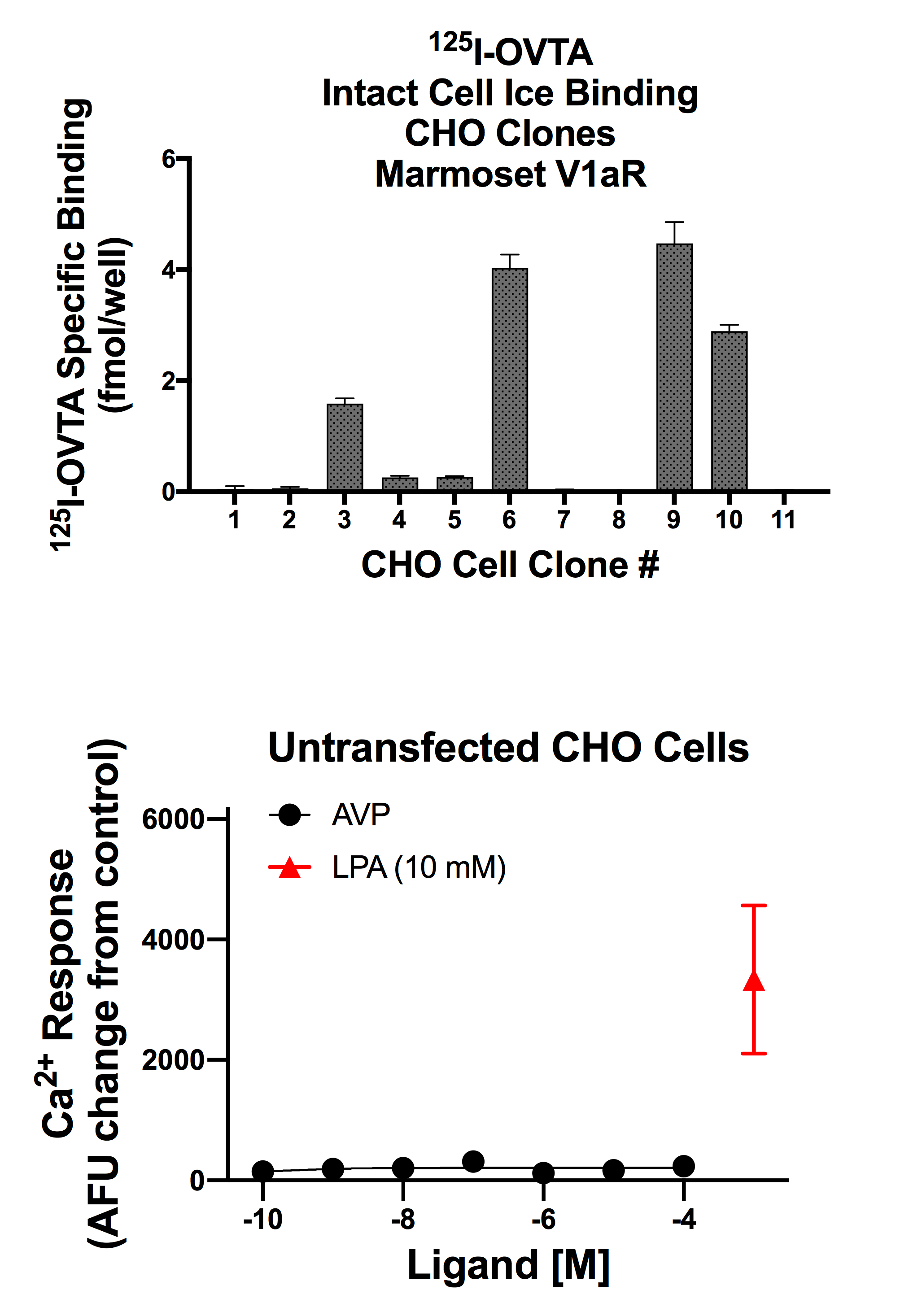
**

**SI Figure 3: Endogenous binding and signaling activity in response to OXT and/or AVP stimulation in CHO cells**. A) ^125^I-OVTA binding activity in clones of CHO cells transfected with marmoset AVPR1a. No ^125^I-OVTA binding responses show clones that have low to no expression of AVPR1a or no to low endogenous OXTR expression. B) untransfected CHO cells show no Ca^2+^ response to AVP (100 mM to 0.1 nM), but do show a Ca^2+^ response to 10 mM Lysophosphatidic acid (LPA) suggesting normal endogenous G-protein activity, but no to low endogenous OXTR or AVPR1a expression.

**
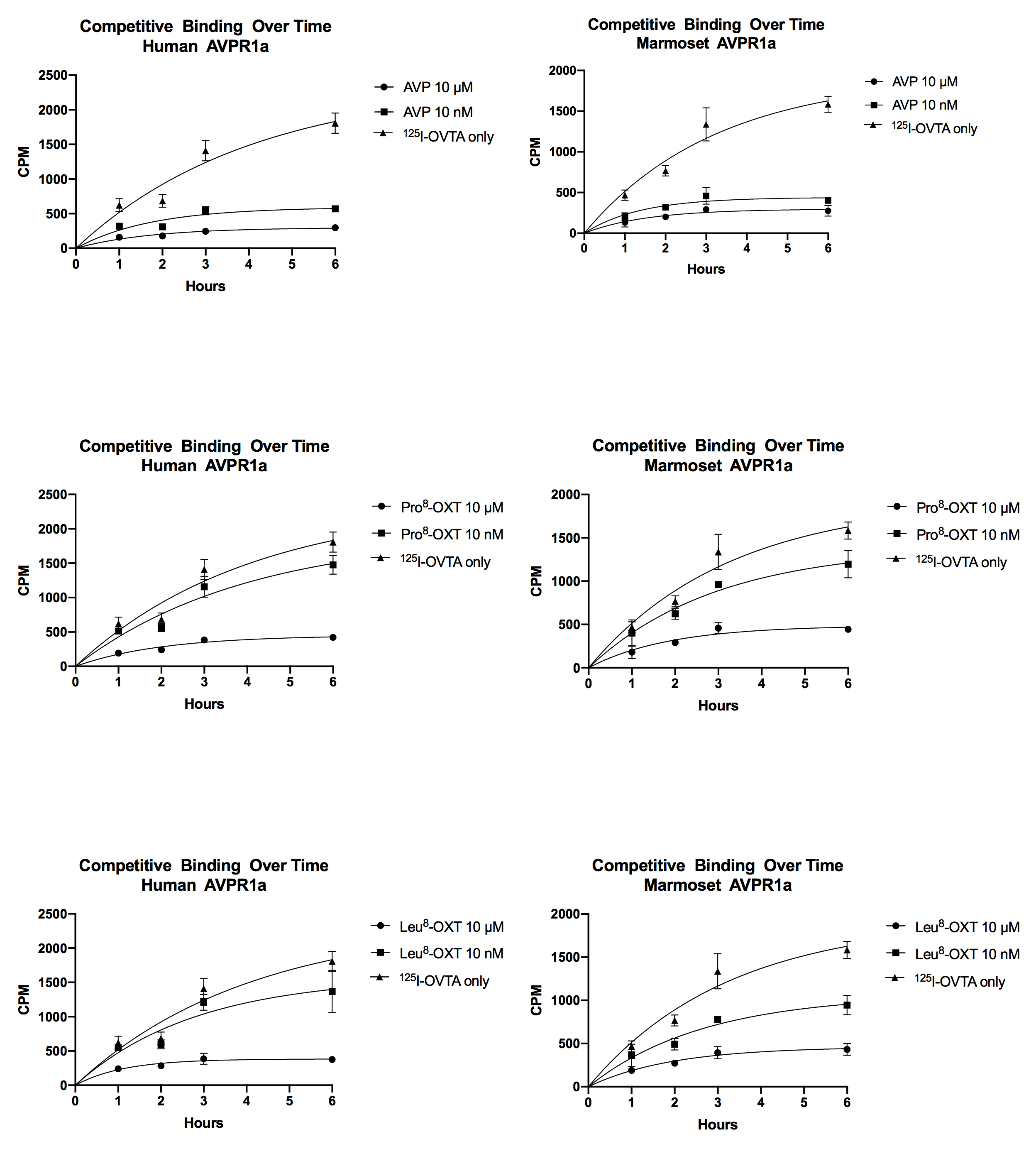
**

**SI Figure 4: Kinetic competitive binding of ^125^I-OVTA with AVP, Leu^8^-OXT, and Pro^8^-OXT in CHO cells expressing human or marmoset AVPR1a**. Incubation on ice for 1, 2, 3, and 6 hours using high (10 μM) and low (10 nM) concentrations of AVP, Leu^8^-OXT, and Pro^8^-OXT with ~ 12 fmol/well of ^125^I-OVTA. CPMs of bound ^125^I-OVTA 3 hr and 6 hr are significantly higher than hour 1 and 2, but not different from each other, suggesting 3 hrs incubation on ice is enough to be at or very near equilibrium for these ligands/receptors.
